# Serological Evidence of Multiple Zoonotic Viral Infections among Wild Rodents in Barbados

**DOI:** 10.3390/pathogens10060663

**Published:** 2021-05-28

**Authors:** Kirk Osmond Douglas, Claire Cayol, Kristian Michael Forbes, Thelma Alafia Samuels, Olli Vapalahti, Tarja Sironen, Marquita Gittens-St. Hilaire

**Affiliations:** 1 Centre for Biosecurity Studies, The University of the West Indies, Cave Hill, St. Michael BB11000, Barbados; 2 Department of Wildlife, Fish, and Environmental Studies, Swedish University of Agricultural Sciences, Skogsmarksgränd 17, 901 83 Umeå, Sweden; claire.c.cayol@gmail.com; 3 Department of Biological Sciences, University of Arkansas, Fayetteville, AR 72701, USA; kmforbes@uark.edu; 4 Epidemiology Research Unit, Caribbean Institute for Health Research (CAIHR), The University of the West Indies, Mona, Kingston 7, Jamaica; alafiasam@gmail.com; 5 Department of Virology, Faculty of Medicine, University of Helsinki, Medicum, Haartmaninkatu 3, 0290 Helsinki, Finland; olli.vapalahti@helsinki.fi (O.V.); tarja.sironen@helsinki.fi (T.S.); 6 Faculty of Medical Sciences, The University of the West Indies, Cave Hill, St. Michael BB11000, Barbados; marquita.gittens@cavehill.uwi.edu; 7 Best–dos Santos Public Health Laboratory, Enmore #6, Lower Collymore Rock, St. Michael BB11155, Barbados

**Keywords:** orthohantavirus, infectious disease, Caribbean, zoonosis, biosecurity

## Abstract

Background: Rodents are reservoirs for several zoonotic pathogens that can cause human infectious diseases, including orthohantaviruses, mammarenaviruses and orthopoxviruses. Evidence exists for these viruses circulating among rodents and causing human infections in the Americas, but much less evidence exists for their presence in wild rodents in the Caribbean. Methods: Here, we conducted serological and molecular investigations of wild rodents in Barbados to determine the prevalence of orthohantavirus, mammarenavirus and orthopoxvirus infections, and the possible role of these rodent species as reservoirs of zoonotic pathogens. Using immunofluorescent assays (IFA), rodent sera were screened for the presence of antibodies to orthohantavirus, mammarenavirus (Lymphocytic choriomeningitis virus—LCMV) and orthopoxvirus (Cowpox virus—CPXV) infections. RT-PCR was then conducted on orthohantavirus and mammarenavirus-seropositive rodent sera and tissues, to detect the presence of viral RNA. Results: We identified antibodies against orthohantavirus, mammarenavirus, and orthopoxvirus among wild mice and rats (3.8%, 2.5% and 7.5% seropositivity rates respectively) in Barbados. No orthohantavirus or mammarenavirus viral RNA was detected from seropositive rodent sera or tissues using RT–PCR. Conclusions: Key findings of this study are the first serological evidence of orthohantavirus infections in *Mus musculus* and the first serological evidence of mammarenavirus and orthopoxvirus infections in *Rattus norvegicus* and *M. musculus* in the English-speaking Caribbean. Rodents may present a potential zoonotic and biosecurity risk for transmission of three human pathogens, namely orthohantaviruses, mammarenaviruses and orthopoxviruses in Barbados.

## 1. Introduction

Rodent species are the most abundant terrestrial animals (representing 43% of mammalian species) found globally and exist as a part of biological ecosystems, however they do pose significant threats to public health [1,2]. The threat of zoonotic infections from wild rodents is well-known, with the risk of infections involving various species of bacteria, fungi, parasites and viruses [1]. Most rodent-borne infections are transmitted through direct or indirect contact with infected rodents and their excreta [1,2]. For this reason, factors such as rainfall, topography, vegetation and occupation, which influence human exposure to rodents or pathogen survival in the environment, have been associated with rodent-borne pathogen infections including *Leptospira*, orthohantaviruses, mammarenaviruses and orthopoxviruses [3,4,5,6,7,8]. Intraspecies transmission of mammarenaviruses, orthohantaviruses and orthopoxviruses have been documented, and occurs following intraspecific wounding, grooming, and with subsequent contact and contamination with infected saliva, excreta and infectious aerosols [9,10,11,12,13].

Orthohantaviruses are single stranded negative-sense RNA viruses, approximately 120–160 nm in diameter from the Orthohantaviridae virus family [14,15,16]. Orthohantavirus infection is an emerging disease throughout the world. Orthohantavirus infection can cause two main clinical diseases, namely haemorrhagic fever with renal syndrome (HFRS) and hantavirus pulmonary syndrome (HPS), or hantavirus cardiopulmonary syndrome (HCPS) [17]. Old World hantaviruses are responsible for causing HFRS and a mild form of HFRS, nephropathica epidemica, whereas New World hantaviruses are responsible for HPS or HCPS. Zoonotic transmission of orthohantaviruses occurs through human contact with infected rodents and/or their excreta or saliva, or indirect contact through inhalation of infectious aerosols contaminated by infected rodent excreta and/or saliva. The natural reservoir hosts of orthohantaviruses include bats, rodents, moles and shrews [18,19,20]. Orthohantavirus circulation in wild rodents can be intriguing, since one rodent species can host more than one orthohantavirus and more than one orthohantavirus can circulate in the same geographical location, with separation influenced by habitat and rodent distribution and range [21,22,23].

*Mammarenaviridae* are a family of single-stranded RNA viruses found in mammals and boid snakes. They are divided into two serogroups based on shared antigens and geographic distribution: (a) Lymphocytic Choriomeningitis-Lassa virus serocomplex viruses, or the Old World arenaviruses, and (b) Tacaribe serocomplex viruses, or the New World arenaviruses, (NWV) [24]. Humans typically become infected with mammarenaviruses through contact with excreta from infected rodents by inhalation of contaminated aerosols, but also via a faecal–oral route of contaminated food, and/or broken skin [9]. In addition, no evidence exists for mammarenavirus infection among rodent species in the English-speaking Caribbean to date.

Cowpox virus (CPXV) is a uniform species virus of the genus *Orthopoxvirus*, family *Poxviridae*, and is antigenically and genetically related to the variola virus, vaccinia virus, and monkeypox virus. It is established as the causative agent of cow pox, a zoonosis causing lesions on the udder of dairy cows and the hands of persons in frequent contact with cows [25,26,27]. CPXV has been detected in a wide host range including voles, rats, cattle, horses, llamas, zoo animals and humans in Europe [27,28]. The reservoir hosts of CPXV are wild rodents, cows, domestic cats, and humans are incidental hosts. Although an orthopoxvirus study among wild songbirds has been conducted in Trinidad, no published studies of CPXV among rodents have been conducted previously in the Caribbean [29].

Given the paucity of data on the zoonotic potential of rodents causing transmission of orthohantavirus, mammarenavirus and orthopoxvirus infections in the Caribbean, a serological study of wild rodents is fully warranted. We report serological evidence of orthohantavirus, mammarenavirus and orthopoxvirus infections among wild rodents, and a molecular investigation of orthohantavirus infection status of three rodent species, *Rattus norvegicus*, *Rattus rattus* and *Mus musculus,* in Barbados. These should provide useful data to aid in the understanding, awareness, control and future prevention of three rodent-borne zoonotic diseases caused by orthohantavirus, mammarenavirus and orthopoxvirus infections in Barbados and the wider Caribbean.

## 2. Results

### 2.1. Wild Rodents Trapping Survey

**Figure 1:**
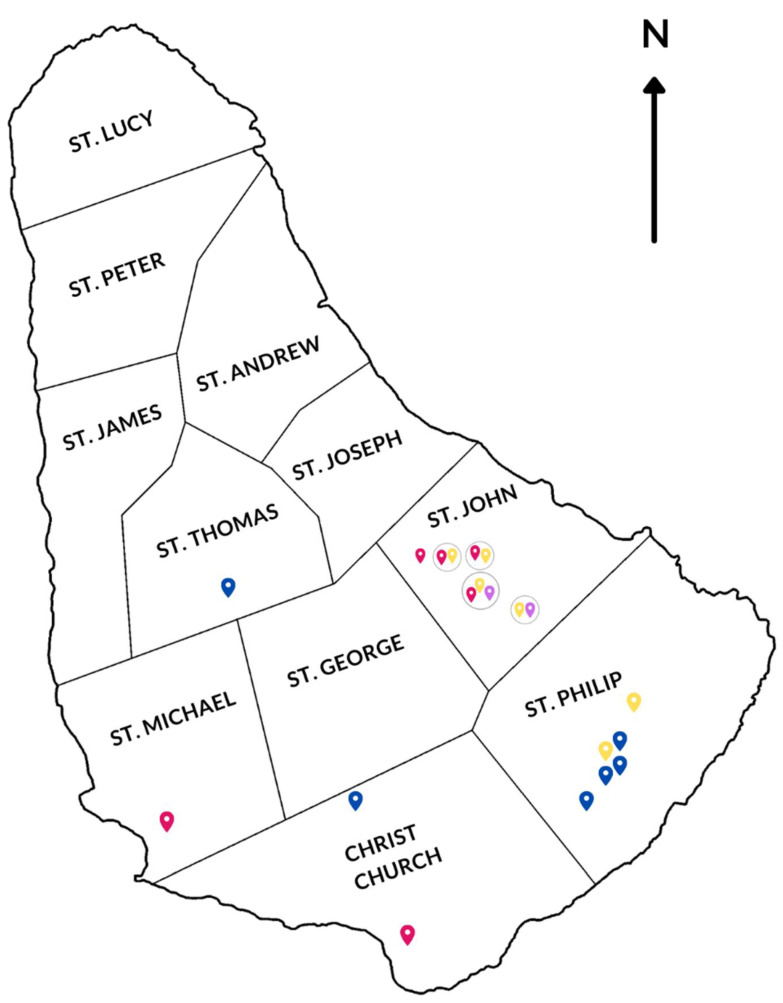
Wild rodent sampling sites in Barbados during the study period (January 2019). Blue location marks indicate wild rodent sampling sites where no orthohantavirus-, mammarenavirus- or orthopoxvirus-seropositive rodents were trapped. Red, purple and yellow location marks indicate sampling areas where orthohantavirus-, mammarenavirus- and orthopoxvirus-seropositive rodents respectively were trapped. Single sampling sites where rodents were found with serological evidence of more than one of the target viral pathogen infections are encircled.

To understand the possible rodent reservoirs of orthohantaviruses, mammarenaviruses and orthopoxviruses in Barbados, a rodent trapping and sampling survey was conducted in 2019. A total of 160 rodents were trapped over 10 trapping nights from 15th January to 26th January 2019, at a total of 15 trapping sites around Barbados including chicken farms, recycling centres, horse stables, an agriproducts retail store, residential neighbourhoods, the national geriatric hospital, and sugarcane fields in parishes where previously recorded human orthohantavirus cases occurred (Table 1 & Figure 1) [30].

Primarily more wild mice (*M. musculus*) were caught in comparison to wild rats (*R. rattus* and *R. norvegicus*) during the trapping survey (Table 1). Three rodent species were trapped, namely *M. musculus* (93.8%, 150/160), *R. norvegicus* (5%, 8/160) and *R. rattus* (1.3%, 2/160) (Table 1). Of the *M. musculus* rodents trapped, 54% (81/150) were males compared to 45.3% (68/150) females, 56.8% (46/81) of the males had developed scrota indicative of reproductive maturity, whilst 43.2% (35/68) did not. Further, 17.6% (12/68) of the females were pregnant, 76.5% (52/68) were non-parous and with 5.9% (4/68) of the female *M. musculus* rodents the reproductive maturity was either indiscernible or not recorded (Table 1).

Of the *R. norvegicus* rodents trapped, 12.5% (1/8) were males compared to 87.5% (7/8) females, 0% (0/1) of the males had developed scrota indicative of reproductive maturity whilst 100% (1/1) did not. Further, 0% (0/8) of the females were pregnant whilst 87.5% (7/8) were non-parous, and with 12.5% (1/8) the reproductive maturity was either indiscernible or not recorded (Table 1). There were only two (2) *R. rattus* rodents, both scrotal males, trapped during this study. Additionally, one rodent was trapped but the observed species identification was inadvertently not recorded.

### 2.2. Orthohantavirus IFA & RT-PCR Testing of Wild Rodents

**Figure 2:**
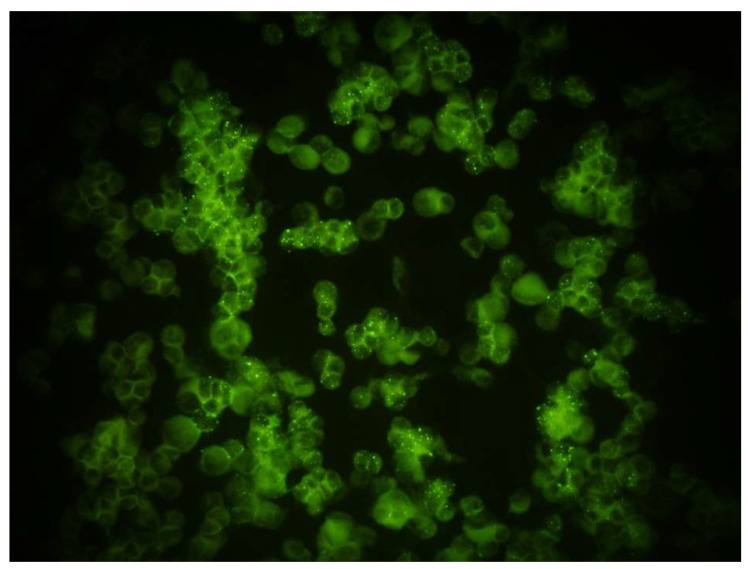
Orthohantavirus [PUUV)] specific IFA IgG testing and staining of a seropositive rodent serum sample.

Dried rodent blood was used to obtain rodent sera in all but two (2) rodents, where hearts were used to obtain the sera. Screening of sera from wild rodents trapped in seven different parishes in Barbados was conducted using IFA to identify seropositive rodents with orthohantavirus-specific IgG antibodies (Table 2, Figure 1 and Figure 2). Of the 160 rodents tested, 3.8% (6/160) were orthohantavirus IFA-positive. For mice, *Mus musculus,* 4.0% (6/150) were orthohantavirus IFA-positive, while for both rat species, *R. norvegicus* and *Rattus rattus,* none of them (0% (0/8) and 0% (0/2) respectively) were orthohantavirus IFA-positive (Table 2). 

The lack of orthohantavirus seropositivity among rats in this study is notable in comparison to a previous rodent survey in Barbados [31]. The orthohantavirus seropositive rodents identified in this current study were trapped at sites in St. John (66.7%, 4/6), St. Michael (16.7%, 1/6) and Christ Church (16.7%, 1/6), including residential neighbourhoods, a local geriatric hospital, sugarcane fields and chicken farms (Figure 1). The two rodent sera (obtained from hearts) were both seronegative for all viral pathogens under investigation.

Tissues and sera from seropositive wild rodents were then tested using orthohantavirus-specific RT-PCR. Molecular investigations failed to detect orthohantavirus vRNA among wild rodents in Barbados, as none of the seropositive rodent sera nor tissues yielded positive orthohantavirus-specific RT-PCR results (Table 2).

Mammarenavirus serosurveys of wild rodents in Barbados and in the Caribbean are non-existent. Thus, screening of wild rodent sera was conducted using mammarenavirus-specific IFA to identify seropositive rodents with orthohantavirus-specific IgG antibodies (Table 2). Of the 160 wild rodents trapped during the study, 2.5% (4/160) were seropositive for mammarenavirus infection using LCMV-specific IFA testing (Table 2). Among *M. musculus* rodents, 2.7% (4/150) were seropositive whilst 0% (0/8) of *R. norvegicus* and 0% (0/2) *R. rattus* rodents were seropositive for mammarenavirus infection (Table 2). All the mammarenavirus-seropositive rodents were trapped in sugarcane fields and at a recycling centre in St. John (Table 2 & Figure 1).

After identification of seropositive wild rodents using IFA, their sera and tissues were screened for mammarenavirus-specific vRNA using RT-PCR (Table 2). None of the seropositive rodent sera nor tissues were positive following mammarenavirus-specific RT-PCR; thus, these molecular investigations failed to detect the presence of mammarenavirus vRNA among wild rodent species in Barbados (Table 2).

### 2.3. Orthopoxvirus IFA Testing of Wild Rodents

Given the re-emergence of CPXV as a zoonotic agent in North America and Europe, and the lack of previous data in Barbados or the Caribbean, a serological investigation of orthopoxvirus infection among wild rodents was conducted. Of the 160 wild rodents sampled, 7.5% (12/160) were seropositive for orthopoxvirus infection (Table 2). For mice, *M. musculus,* 6.7% (10/150) were orthopoxvirus IFA-positive, whilst for both rat species, *R. norvegicus* and *R. rattus,* 12.5% (1/8) and 50% (1/2) respectively were seropositive following orthopoxvirus IFA testing (Table 2). Among the orthopoxvirus-seropositive rodents trapped in the study, 6.5% (6/92) were trapped in St. John and 14% (6/43) in St. Philip respectively, from sugarcane fields, chicken farms, residential neighbourhoods and a recycling centre (Table 2 & Figure 1).

## 3. Discussion

Orthohantavirus, mammarenavirus and orthopoxvirus studies on rodents in Caribbean countries have been sparse or non-existent, and we present the first serological evidence of multiple orthohantavirus, mammarenavirus and orthopoxvirus infections in wild rodent species in both Barbados and the English-speaking Caribbean. Seroprevalence rates of 3.8% for orthohantavirus, 2.5% for mammarenavirus and 7.5% for orthopoxvirus infections were observed among wild rodents in Barbados during this study. This has potential biosecurity implications for farms, hospitals, residential homes, businesses involved in recycling and persons working in and living around sugarcane fields.

The last report of orthohantavirus infection among wild rodents in Barbados was in 2002, thus more updated data were required to understand the orthohantavirus infection status of wild rodents [31]. This study included wild *M. musculus* rodents to test the hypothesis of *M. musculus* as possible rodent vectors of orthohantavirus infections in Barbados and represents the first serological evidence of orthohantavirus infection of *M. musculus* in the Caribbean and the first evidence of mammarenavirus infection and orthopoxvirus infection among wild rodents in the Caribbean.

### 3.1. Orthohantavirus Infection among Wild Rodents

Given the known rodent hosts present in Barbados, *Rattus* and *Mus* spp., SEOV infections would be expected given its worldwide distribution [32]. Orthohantavirus prevalence among wild rodents varies by geographic location from 2.5% in Brazil, 5.6% to 7.7% in Paraguay, 4.6% to 5.6% and as high as 23.5% in Panama, and even higher rates of 29% and 28% in Grenada and Barbados respectively [31,33,34,35,36,37]. The overall orthohantavirus seropositivity rate of 3.8% observed among all rodents in this study, and among *R. norvegicus* (0%) and *M. musculus* (4%), were like those observed in the USA, Kuwait, China and Europe [38,39,40,41]. The lack of orthohantavirus seropositivity among wild rats in this study may be due to the low numbers of rats trapped and the low percentage of males, as adult male rats are known to have higher orthohantavirus seroprevalence rates [13]. Orthohantavirus seropositive *M. musculus* rodents were identified by IFA testing, highlighting the possible risk these may play in orthohantavirus transmission in Barbados. The lack of genetic evidence of orthohantavirus infection among wild rodents, and the lack of demonstration of onward transmission, limits the conclusion of their potential as orthohantavirus reservoirs in Barbados. Limited orthohantavirus research has been conducted in the Caribbean. The first serological evidence of orthohantavirus infections in the Caribbean involved the detection of anti-orthohantavirus antibodies in suspected leptospirosis patients and rodents in Barbados [31]. In this study, 12% of 60 patients presenting with febrile illness possessed orthohantavirus-specific immunoglobulin M (IgM) [31]. The identity of the circulating orthohantavirus strain(s) and their source in Barbados has remained unknown. Evidence of Maporal, Caño Delgadito and Araraquara orthohantavirus infections in rodents, and the presence of multiple rodent hosts in Venezuela, Brazil and Columbia [42,43,44,45] along with a recent HPS outbreak in French Guiana in 2016, enhance the risk of new and more lethal orthohantavirus strains entering the Caribbean region via international and inter-regional trade and travel [46].

Orthohantavirus transmission is influenced by environmental and climatic factors including rainfall, topography and vegetation [3,4,47]. High rainfall can be associated with increased orthohantavirus transmission, as higher infection rates were observed during the wet season compared to the dry season since rainfall can permit moist soil which facilitates rodent burrowing, breeding, survival and the proliferation of vegetation and food for rodents [4,47]. The rainy season in Barbados has been associated with higher human orthohantavirus prevalence rates, with peaks occurring in the months of August and September [30]. A lower orthohantavirus seroprevalence of 3.8% was observed in this study compared to the previous rodent study, with a 29% seroprevalence rate. This may be due to a few differences between the former and currently reported rodent serosurveys, namely (1) sample size, 68 rodents vs. 160 rodents, (2) the proportion of rodent species, predominantly *Rattus* spp. and no *M. musculus* rodents vs. predominantly *M. musculus* rodents, and (3) timing of sampling, wet season (July–August 2000) vs. dry season (January 2019) [31]. These differences may have resulted in the lower orthohantavirus seroprevalence rate observed, and the lack of detection of orthohantavirus vRNA among wild rodents.

Conversely, excessive rainfall and/or extreme weather events including flooding can result in the reduction of rodent populations, reduced risk of orthohantavirus transmission, and reduced orthohantavirus seroprevalence [48]. However, no major flooding events were recorded during 2018 in Barbados. Other climatic factors influencing orthohantavirus transmission include atmospheric moisture variability and temperature [48], so tropical climatic conditions such as high temperature and humidity in Barbados could influence the survival of orthohantaviruses in the environment and their transmission. Further research on the influence of abiotic factors in orthohantavirus transmission is therefore necessary to understand the orthohantavirus ecology in Barbados and the Caribbean. Future studies to obtain genetic evidence of the orthohantavirus strain(s) present are necessary to determine the true reservoir status of rodents, and perhaps should be conducted in synch with peak periods of human orthohantavirus infections to permit higher chances of obtaining genetic sequences from rodents. The inclusion of bats in future rodent serosurveys should also be strongly considered given the detection of orthohantaviruses in various regions around the world [49,50,51,52,53].

### 3.2. Mammarenavirus IFA Seropositive Rodents

Evidence of mammarenavirus infection in rodents, bats and humans within the Americas has been previously documented in the USA, Columbia, Brazil and French Guiana [54,55,56,57,58,59,60,61,62,63,64,65]. A mammarenavirus seroprevalence rate of 2.5% was observed among wild rodents in Barbados during this study, which is comparable to seroprevalence rates observed in wild rodents from Turkey (2.4%), Italy (5.6%), the United Kingdom (5.8%) and the USA (3.1%) [66,67,68,69]. However, the same issues which may have impacted orthohantavirus seroprevalence rates in these wild rodents could have also influenced observed mammarenavirus seroprevalence rates, as both viruses share common routes of intra-species and zoonotic transmission via infected rodent urine and faeces, skin abrasions or cuts, and the inhalation of dust or aerosols contaminated by rodent excreta.

The specific mammarenavirus infecting *M. musculus* in this study is unknown, as antibody cross-reactivity can be observed among mammarenaviruses and only FRNT and genetic evidence can confirm the identity of mammarenaviruses [70,71]. One possible mammarenavirus that may be present among wild *M. musculus* in Barbados is LCMV, which is a teratogen causing birth defects when acquired by pregnant mothers. Though LCMV has a world-wide distribution, viruses of the LCM-LAS complex are found primarily in Africa, whereas the Tacaribe complex viruses are found in North and South America. The Tacaribe complex includes the following viruses: Tacaribe, Allpahuayo, Amapari, Bear Canyon Virus, Chapare, Cupixi, Flexal, Guanarito, Junin, Latino, Machupo, Oliveros, Paraná, Pichinde, Pirital, Sabiá, Tamiami, and Whitewater Arroyo.

Mammarenavirus studies in the Caribbean, however, have been limited even though the Tacaribe virus has been isolated since the 1950s in Trinidad and Tobago, and some virological studies conducted with Jamaican fruit bats (*Artibeus jamaicensis*) and mosquitoes have discovered the presence of this virus in Trinidad and Tobago [57,72]. The role of *A. jamaicensis* as a possible reservoir host was explored and found both to be susceptible to infection by Tacaribe virus and to exhibit disease pathology, suggesting this species may not be the reservoir for Tacaribe virus [73]. This virus was also isolated from lone star (*Amblyomma americanum*) ticks in Florida, USA, and as this tick is known to feed on humans, the ability of the lone star tick to transmit this virus to people requires further investigation [74]. At present, the reservoir host of the Tacaribe virus remains unknown. Future studies should explore the source of mammarenavirus infections in wild rodents.

### 3.3. Orthopoxvirus IFA Seropositive Rodents

We also report the first serological evidence of orthopoxvirus infection among wild rodents in the Caribbean, with an orthopoxvirus seroprevalence rate of 7.5% observed among wild rodents. This is on the lower end of orthopoxvirus seroprevalence rates observed compared to studies of wild rodents in Europe and Africa, including Finland (33%), Germany (32%), Uganda (42%), Hungary (18%), Turkey (0.3%) and Serbia (3.2%) [67,75,76,77]. Evidence of orthopoxvirus infection outside of Europe is limited, and thus this adds to the limited knowledge of orthopoxvirus infections worldwide and is of importance, as orthopoxviruses continue to be emerging zoonotic agents [28,78,79]. In Europe, bank voles (*Clethrionomys glareolus*) and wood mice (*Apodemus sylvaticus*) constitute the main reservoirs [27], whereas CPXV was sporadically detected in rats (*Rattus norvegicus*) [80,81]. Domestic cats can play a role in the transmission of CPXV to humans [82,83]. Direct transmission of CPXV from rodents to humans has also been documented, including rats and mongoose [81,84,85,86,87]. CPXV infection of immunocompetent persons usually results in localized lesions mainly on fingers, hands, or the face [88]. However, in immunocompromised patients, severe generalized CPXV infections have been documented [89,90].

The lack of smallpox vaccinations in the late 1970s, and an increasing trend of keeping wildlife as pets, have been offered as the reasons for increasing human cases of smallpox in Europe [28,81,86]. Public health education regarding the zoonotic pathogens potentially transmissible by rodents (mice and rats) in Barbados can be updated to include these zoonotic viral pathogens, and biosecurity awareness training given to at-risk persons including cow farmers, dairy workers, ancillary staff, sanitation workers, veterinarians, and the general public.

### 3.4. Strengths

Several key findings were observed in this study, including (1) the identification of a new rodent species in the Caribbean, *M. musculus*, with serological evidence of orthohantavirus infection and (2) serological evidence of multiple viral human pathogens, namely orthohantavirus, mammarenavirus and orthopoxvirus infections among wild rats and mice, *R*. *norvegicus* and *M*. *musculus* respectively, in Barbados.

### 3.5. Study Limitations

This study had some limitations and would benefit from some improvements, including (1) larger sample sizes for rodents (particularly rats), (2) a change in the timing of rodent sampling which ideally should coincide with a variation in rainfall and climatic conditions (dry and wet seasons) and preferably over multiple years and (3) the absence of sampling of other possible virus zoonosis reservoirs such as bats [49,53,91].

### 3.6. Recommendations

To limit the potential exposure to viral pathogens from wild rodents, enhanced public awareness should be conducted to inform persons of potential transmission risks of mammarenavirus, orthohantavirus and orthopoxvirus infections and to improve biosecurity at residences, on farms, in hospitals and in other places of business.

## 4. Materials and Methods

### 4.1. Rodent Trapping and Sample Collection

A prospective cross-sectional wild rodent survey was conducted during January 2019 to investigate the serological and molecular evidence of orthohantavirus, mammarenavirus and orthopoxvirus infection status. In total, 160 rodents were trapped using Sherman and Tomahawk traps at 15 different urban, farm and rural locations around Barbados (Figure 1). Traps were baited with peanut butter, banana, bacon and English potato (for a source of moisture for the trapped animal). Care was taken to ensure the traps were shaded to minimize heat exhaustion of trapped rodents. Traps were collected the following morning after being set the preceding afternoon/evening. Trapping was conducted for at least two nights per site with a minimum of 8 traps per location. Trapped rodents were euthanized by chloroform inhalation on a saturated cotton wad followed by cervical dislocation. For each rodent, its species, sex, and biometrics (weight, tail and body length) were recorded.

Dissections were conducted to collect tissue samples (liver, kidney, spleen, heart, lung and ear). Blood samples were obtained from the body cavities by allowing the blood to be absorbed in the absorption zones of pre-cut Nobuto blood filter papers. The filter papers with blood were allowed to dry completely before being wrapped in tissue paper and stored in a cool, dark place. After drying and storing the filter papers, a 1 cm^2^ piece of the absorption zones of the filter papers with rodent blood was cut, diluted with 350 μL of Dulbecco medium + 0.2% bovine serum albumin (BSA) (pH 7.2) and shaken overnight with gentle rocking at 4 °C, to elute the blood sample. In the absence of a filter paper blood sample, the heart was used, and no dilution was necessary. The eluate was then tested using the respective virus-specific IFA.

### 4.2. Serological Testing of Rodent Sera

Each rodent blood sample was screened by indirect IFA for orthohantavirus infection using three separate orthohantavirus strain antigens (PUUV, SEOV and HTNV) with infected and non-infected cells mixed in a ratio of 1:3, to ensure specificity of the readouts as described previously (Figure 2) [67]. For each rodent sample, screening was done for orthohantavirus- (SEOV and PUUV), mammarenavirus- (LCMV) and orthopoxvirus- (CPXV)-specific IFA, by placing 20 uL of each sample on the respective IFA slide well. For negative controls, 20 uL of PBS was added (along with anti-mouse IgG). For the positive control well, 20 uL of the respective positive control sera was added (SEOV- or PUUV-positive human sera (1/20 dilution) for orthohantavirus, LCMV-positive mouse monoclonal antibody (1/2 dilution) and CPXV-positive human sera). The slides were then incubated in the moist chambers for 30 min at 37 °C. Each slide was rinsed with cold PBS (4 °C) solution to remove excess antibodies. This was repeated 3 times with cold PBS for 5 min each. The final rinse was performed in double distilled water (ddH_2_O) for 5 min and air dried with a fan. Then 20 µL of polyclonal rabbit anti-mouse fluorescein isothiocyanate (FITC) conjugate (diluted 1:100), or polyclonal sheep anti-rat FITC conjugate (diluted 1:2000) was placed in the rodent (mouse or rat) sample wells, anti-mouse or anti-rat negative control well and LCMV positive control well, or orthohantavirus positive control well. A 20 µL aliquot of anti-human IgG FITC conjugate (diluted 1:100) was added to the human samples, anti-human negative control, and positive control wells. The slides were incubated in the moist chamber for 30 min at 37 °C, washed 3 times with cold PBS washing solution, then rinsed once with ddH_2_O to remove excess conjugate and air dried using a fan. The slides were protected from light and stored at 4 °C until reading with a UV microscope. After observation of slides using the microscope, a note of seropositivity or negativity was recorded for each sample, as well as any unspecific or unclear reactions. Reciprocal titres of >16 by IFA were considered positive results.

### 4.3. RNA Extraction and Molecular Testing of Rodent Sera and Tissue

Lung and kidney samples from seropositive rodents were lysed and homogenized using Roche MagNA lyser (Basel, Switzerland) and Trizol^™^ reagent (ThermoFisher Scientific, Waltham, MA, USA), and RNA was extracted following the manufacturer’s instructions. RNA yield was determined using NanoDrop (ThermoFisher Scientific, Waltham, MA, USA) based on absorbance measurements at 260 nm and 280 nm. Pan-orthohantavirus and mammarenavirus RT–PCRs were conducted as previously described [92,93].

## Figures and Tables

**Table 1:** Description of wild rodents trapped in Barbados during January 2019.

		Gender and Reproductive Status					
		Male			Female		
No. of Trapped Rodents	Rodent Species	N	SC	Total	PR	NP	Total
150 *^,+^	*Mus musculus*	35	46	81	12	52	64
8 *	*Rattus norvegicus*	1	0	1	-	6	6
2	*Rattus rattus*	-	2	2	-	-	0
160		36	48	84	12	58	70

Key: N—non-scrotal male; SC—scrotal; PR—pregnant; NP—non-parous; *—with 4 female *Mus musculus* and 1 female *Rattus norvegicus* rodents, their reproductive status was either indiscernible or inadvertently not recorded. ^+^—With one (1) *Mus musculus* rodent, the observed gender identification was not recorded.

**Table 2:** Serological and molecular survey study of wild rodents in Barbados.

Rodent Species	No. of Trapped Rodents	Trapping Location	IFA Testing			RT-PCR	
			Orthohantavirus (PUUV)	Mammarenavirus (LCMV)	Orthopoxvirus (CPXV)	Orthohantavirus	Mammarenavirus
*Rattus rattus*	2	St. John	0/2	0/2	1/2	-	-
*Rattus norvegicus*	8	St. Philip	0/8	0/8	1/8	-	-
*Mus musculus*	5	St. Michael	1/5	0/5	0/5	0/1	-
	3	St. Thomas	0/3	0/3	0/3	-	-
	90	St. John	4/90	4/90	5/90	0/4	0/4
	35	St. Philip	0/35	0/35	5/35	-	-
	7	Christ Church	1/7	0/7	0/7	0/1	-
	160		6/160 (4.0%)	4/160 (2.5%)	12/160 (7.5%)		

N.B. There were 15 sites where wild rodents were trapped in 7 different parishes, including sugarcane fields, recycling centres, horse stables, a national geriatric hospital, chicken farms, an agriproducts retail store and residential neighbourhoods. Key:—no testing necessary; PUUV (Puumala orthohantavirus); LCMV (lymphocytic choriomeningitis virus); CPXV (cowpoxvirus); IFA (immunofluorescent assay); RT-PCR reverse transcriptase polymerase chain reaction 2.3. Mammarenavirus IFA & RT-PCR testing of wild rodents.

## Data Availability

The GPS locations of the geographical locations of rodent trapping sites during the study in Barbados were recorded and illustrated using Google Maps. https://www.google.com/maps/d/u/1/edit?mid=1HJoJ2emo-HGoU96ziA1q-vALuRgM9b_3&ll=13.289998404811126%2C-59.605584127734375&z=11 accessed on 27 May 2021.
